# Perception, Attitude, and Confidence of Physicians About Antimicrobial Resistance and Antimicrobial Prescribing Among COVID-19 Patients: A Cross-Sectional Study From Punjab, Pakistan

**DOI:** 10.3389/fphar.2021.794453

**Published:** 2022-01-04

**Authors:** Khezar Hayat, Zia Ul Mustafa, Muhammad Nabeel Ikram, Muhammad Ijaz-Ul-Haq, Irum Noor, Muhammad Fawad Rasool, Hafiz Muhammad Ishaq, Anees Ur Rehman, Syed Shahzad Hasan, Yu Fang

**Affiliations:** ^1^ Department of Pharmacy Administration and Clinical Pharmacy, School of Pharmacy, Xi’an Jiaotong University, Xi’an, China; ^2^ Institute of Pharmaceutical Sciences, University of Veterinary and Animal Sciences, Lahore, Pakistan; ^3^ Center for Drug Safety and Policy Research, Xi’an Jiaotong University, Xi’an, China; ^4^ Shaanxi Centre for Health Reform and Development Research, Xi’an, China; ^5^ Department of Pharmacy Services, District Headquarter (DHQ) Hospital, Pakpattan, Pakistan; ^6^ Department of Surgery, Faisalabad Medical University, Faisalabad, Pakistan; ^7^ Hamdard Institute of Pharmaceutical Sciences, Hamdard University, Islamabad, Pakistan; ^8^ Department of Pathology, Quaid-e-Azam Medical College, Bahawalpur, Pakistan; ^9^ Department of Pharmacy Practice, Faculty of Pharmacy, Bahauddin Zakariya University, Multan, Pakistan; ^10^ Faculty of Veterinary and Animal Sciences, Muhammad Nawaz Shareef University of Agriculture, Multan, Pakistan; ^11^ Department of Pharmacy, University of Huddersfield, Huddersfield, United Kingdom

**Keywords:** antimicrobial resistance, COVID-19, coronavirus, antibiotics, physicians

## Abstract

**Background:** Patients with coronavirus disease 2019 (COVID-19) could experience multiple coinfections, and judicial antimicrobials, including antibiotics, is paramount to treat these coinfections. This study evaluated physicians’ perception, attitude, and confidence about antimicrobial resistance (AMR) and antimicrobial prescribing in patients with COVID-19.

**Methods:** A self-administered and validated online questionnaire comprised of six sections was disseminated among physicians working in public sector hospitals in Punjab, Pakistan, using the convenience sampling method from April to May 2021. The study also assessed the validity and reliability of the study questionnaire using exploratory factor analysis and Cronbach’s alpha. In addition, the descriptive and inferential statistics present survey results.

**Results:** A total of 387 physicians participated in this study. The study showed that the questionnaire demonstrated good internal consistency (Cronbach’s alpha = 0.77). Most physicians (*n* = 221, 57.1%) believed that AMR is a considerable problem in Pakistan. Less than a quarter of respondents (*n* = 91, 23.5%) consulted with local antibiotic resistance data to prescribe antibiotics in COVID-19 patients. However, the respondents were confident to select a suitable antibiotic (*n* = 229, 59.2%). More than three-quarters of the respondents believed that advice from a senior colleague (*n* = 336, 86.8%), infectious disease (ID) physician (*n* = 315, 81.4%), and implementing antimicrobial stewardship programs (ASPs) could facilitate appropriate prescribing of antibiotics in COVID-19 patients. Multivariate logistic regression revealed that physicians with more than 10 years of experience had higher odds of consulting local guidelines for antibiotic therapy (OR, 4.71 95% CI: 1.62–13.73, *p* = 0.004) than physicians with less than 5 years of experience. Similar trends were found for consulting national guidelines and local resistance data to select an empiric antibiotic therapy.

**Conclusion:** AMR-related awareness was optimal among physicians. Only a few physicians looked up local antibiotic resistance data before prescribing antibiotics to COVID-19 patients empirically. The significant approaches advised by physicians to reduce AMR risk among COVID-19 patients were the implementation of ASPs combined with advice from ID physicians.

## Introduction

Coronavirus disease 2019 (COVID-19) has invaded nearly every continent globally, with around 261 million laboratory-confirmed cases and 5.2 million deaths as of November 29, 2021 (WHO, 2021). COVD-19 is a communicable disease caused by severe acute respiratory syndrome coronavirus 2 (SARS-CoV-2), a global health threat or a pandemic declared by the World Health Organization (Muralidar et al., 2020). Most of the (∼80%) cases of COVID-19 show symptoms of dry cough, nasal congestion, mild fever, headache, and sore throat. During the first wave of COVID-19, around 5% of cases develop the critical disease with multiple organ injuries and consequent deaths in almost half of these cases (Samudrala et al., 2020).

Since the emergence of the COVID-19 pandemic, many treatment options are being employed to treat infected individuals, including antiviral drugs (remdesivir alone and baricitinib with remdesivir), steroids (dexamethasone), immunosuppressants (tocilizumab), monoclonal antibodies (casirivimab and imdevimab), and convalescent plasma (Jean et al., 2020; Trivedi et al., 2020). However, apart from a viral infection that produces COVID-19, the chances of life-threatening secondary bacterial infections have been increased (Sharifipour et al., 2020). In addition, the severity of the disease among hospitalized patients may worsen the condition, leading to over-prescription of antibiotics and antimicrobial resistance (AMR) (Beović et al., 2020). Therefore, the prescription and administration of antibiotics during the ongoing pandemic were reported to be enhanced significantly (Grau et al., 2021a; Grau et al., 2021b).

Antibiotics are not recommended for viral infections like SARS-CoV-2; their excessive utilization has been observed throughout the world during the initial waves of the COVID pandemic (Strathdee et al., 2020). For example, the European region has reported inappropriate antibiotic use, where 79–96% of the population consumes antibiotics to prevent infection (WHO, 2020b). Early studies from China reported that 58% of hospitalized patients of COVID-19 were prescribed antibiotics (Guan et al., 2020). In Latin American countries, the prevalence of bacterial coinfection among COVID-19 individuals ranges from 3.5 to 6.9%, and 27–84% among patients on antibiotic therapy (Álvarez-Moreno et al., 2021). Therefore, excessive, unjustified, and irrational use of antibiotics in the current pandemic could negatively affect the ongoing antimicrobial stewardship activities leading to an increased risk of AMR (Getahun et al., 2020). Other causes of AMR during the pandemic were disruptions of health care services, vaccination facilities, and ongoing treatment of other infectious diseases like *tuberculosis* and human immunodeficiency virus.

According to the United States Center for Diseases Control and Prevention (CDC), AMR costs more than 20 billion dollars annually with a loss of 35 billion dollars productivity (CDC, 2013). In addition, AMR claims more than 700,000 deaths annually, with a 10-fold increase expected by 2050 with the loss of 1 trillion dollars (Neill, 2014). More than 35,000 deaths are documented in the United States due to AMR, with more than 2.8 million cases of resistance (CDC, 2020).

AMR in low- and middle-income countries (LMICs) is already widespread with limited surveillance data. Recognizing individuals with viral and bacterial infections is challenging due to the lack of cost-effective, readily available biomarkers for such quick differentiation (Do et al., 2021; Lucien et al., 2021). This overuse of antibiotics may possess the risk of 10% increased AMR for several classes of available antibiotics compared with the previous year (Arshad et al., 2020).

Before COVID-19 emergence, AMR was stated as a public health threat by the World Health Organization (WHO), and a massive allocation of resources was made available to halt its pace (WHO, 2015). Global Action Plan (GAP) proposed by the WHO in 2015 to combat AMR consisted of five core objectives: improving AMR awareness, strengthening knowledge through surveillance, infection prevention improvement, allocating necessary sources, and optimizing antimicrobial use agents (WHO, 2020a).

As active and leading members of the health care provision team, physicians are the frontline healthcare professionals providing their services during this pandemic to treat hospitalized COVID-19 patients. Their role could be crucial while selecting and prescribing an appropriate antibiotic to their patients. Pakistan is an LMIC located in South Asia, surrounded by the epicenters of COVID-19 like Iran and India. According to the National Disaster Management Authority (NDMA), about 1.28 million positive cases have been reported, with 28,709 deaths as of November 29, 2021 (NDMA, 2021). However, to the best of our knowledge, no research has been done among Pakistani physicians to evaluate their awareness about antimicrobial prescribing and resistance in COVID-19 patients. Therefore, our study aims to assess Pakistani physicians’ perception, attitude, and confidence about antimicrobial prescribing and resistance among COVID-19 infected patients.

## Methods

### Study Design and Population

A cross-sectional survey was performed using a self-administered and validated questionnaire to address this key issue in the Punjab Province of Pakistan (Sedgwick, 2014). Currently, Punjab Province is home to more than half of Pakistan’s population and consists of nine administrative divisions and thirty-six districts. Compared with other provinces, it has state-of-the-art and well-equipped medical facilities, including hospitals (*n* = 389), dispensaries (*n* = 12,086), child welfare, and maternity centers (*n* = 286), with a bed capacity (*n* = 60,386) (Statistics, 2019).

We approached physicians who were practicing in public sector hospitals and managing COVID-19 patients. Other physicians, interns, and medical students were excluded.

### Development of Study Questionnaire

The development of the study questionnaire involved a detailed review of tools used in previous relevant studies, intending to replicate them, wherever possible, to aid in the comparison of the results (Labricciosa et al., 2018; Hayat et al., 2019a; Hayat et al., 2019b; Beović et al., 2020; Buehrle et al., 2020; Hayat et al., 2020). An initial version of the questionnaire was reviewed by a multidisciplinary team (two academic staff (PhD in Pharmacy) and ten physicians) to determine the content and face validity of the questionnaire. Subsequent changes were made upon receiving their feedback. The questionnaire contained information regarding physicians’ demographics (gender, age, field experience, current working position, and practice area) and COVID-19 related treatment guidelines, training, and resistance data. Five questions were related to physicians’ perceptions about the importance of the problem of AMR. A five-point Likert scale (strongly agree to strongly disagree) was used to rate each question. Twenty-three questions related to participant attitudes during antibiotic prescribing in COVID-19 patients, the use of empirical antimicrobial treatment with activity against specific pathogens, and participants’ choice of antibiotics were asked. Seven questions relating to participants’ confidence about antibiotic prescribing, measured on a scale from very confident to very unconfident. Nine questions about approaches for optimal antibiotic prescribing in COVID-19 patients, which were recorded on a five-point Likert scale.

### Sample Size and Sampling Procedure

A total of 1,25,734 medical doctors are registered in Punjab province with Pakistan Medical Commission (PMC) who perform their duties in several hospital settings (Commission, 2020). The sample size for this study was 383, calculated through the Raosoft online calculator, by considering the margin of error of 5%, with a 95% confidence interval and 50% response distribution. In addition, convenience sampling was utilized to recruit study participants.

Due to the COVID-19 outbreak sweeping the country, an online version of the study questionnaire was used. A web-based online questionnaire (https://forms.gle/ajeTcPLNEmsk9okA8) was developed, and the link to this survey was distributed to the physicians via numerous medical networking platforms, including WhatsApp and Facebook. Before completing the questionnaire, information related to the aim of the study, eligibility criteria, data privacy, right to pull out from participation, voluntary involvement, and consent was provided.

### Statistical Analysis

The Statistical Package for the Social Sciences (SPSS Inc., version 18, IBM, Chicago, IL, United States) was used to analyze all study data with *p* < 0.05 as a level of statistical significance. The descriptive statistics were used to present frequency, percentages, mean, standard deviation, and median. Exploratory factor analysis was performed using Principal Component Analysis (PCA) with Kaiser normalization to examine the construct validity of the study questionnaire. Kaiser-Meyer-Olkin test (KMO) was used to assess sampling adequacy of >0.7. Next, we used Bartlett’s test of sphericity to assess significant correlations between variables. We have also examined the corrected item-total score correlations and internal consistency using Cronbach’s alpha. A Chi-squared test was also applied for categorical variables. The total, average, and summed scores were generated for each domain of the questionnaire. Independent *t*-test and one-way analyses of variance (ANOVA), including post hoc tests, were computed to examine differences in various domains scores and gender, age, and year of experience. Logistic regression analysis with a backward stepwise approach was used to identify the significant factors predicting agreement or disagreement with questionnaire domains. The model was selected based on the model summary with Hosmer and Lemeshow test.

### Validity and Reliability of Study Questionnaire

A 29-items questionnaire was entered into iterated PCA with Kaiser normalization. We examined the sample adequacy using the KMO test prior to factor extraction, which resulted in an overall index of 0.81, suggesting that the sample was adequate for factor analysis. The inter-correlation matrix was found to be factorable based on Bartlett’s test for sphericity (Chi-Square (406) = 6,925.6, *p* < 0.001). Based on the number of factors, a five-factor solution was selected. The loadings of individual items on these three factors are presented in Supplementary Table S1 (Supplementary File). The five underlying factors were 1) perception about antimicrobial resistance amid COVID-19 pandemic, 2) empirical antimicrobial treatment against the specific pathogen in COVID-19 patients, 3) confidence about prescribing an antibiotic in COVID-19 patients, 4) attitude towards measures to improve antibiotic prescribing in COVID-19 patients, and 5) attitude towards the antibiotic prescribing process in COVID-19 patients. The total variance explained by the five dimensions was 63%. An analysis of the individual items showed that the respondents tended to answer all items, and the items were well correlated. The number of items, reliability, and number of respondents for each category are presented in Table 1. The overall reliability (measured as Cronbach’s alpha) value of the scale was 0.77.

**TABLE 1:** Number of items and respondents, reliability, and score distributions by domain included in the Perception and Attitudes of Physicians towards prescribing antimicrobials questionnaire.

Questionnaire domains	Number of items	Scale (possible range)	Number of respondents	Reliability coefficients	Mean (SD)
Empirical antimicrobial treatment in COVID-19 patients	6	Strongly agree to Strongly disagree (1–5)	387	0.68	2.53 (1.31)
Perception about antimicrobial resistance amid COVID-19 pandemic	4	Strongly agree to Strongly disagree (1–5)	387	0.67	1.44 (0.68)
Confidence about prescribing an antibiotic in COVID-19 patients	7	Very confident to very unconfident (1–4)	387	0.94	1.45 (0.53)
Attitude towards measures to improve antibiotic prescribing in COVID19 patients	9	Strongly agree to Strongly disagree (1–5)	387	0.91	2.13 (1.18)
Attitude towards the antibiotic prescribing process in COVID-19 patients	3	Yes or No (1–2)	387	0.79	-
Total	29	-	387	0.77	1.89 (0.92)

## Results

Out of the 700 physicians requested to participate in the study, 387 accepted and completed the questionnaire, with a response rate of 55.3%. The majority of the physicians were females (*n* = 234, 60.5%), aged 25–35 years (*n* = 328, 84.8%), and had an experience of fewer than 5 years (*n* = 219, 56.6%). Most of the physicians (*n* = 290, 74.9%) worked in tertiary care health settings with a capacity of more than 400 beds (*n* = 251, 64.9%). As reported by study respondents, local institution-based treatment guidelines for COVID-19 patients were lacking in most healthcare settings (*n* = 268, 69.3%). Furthermore, less than a quarter of respondents (n = 78, 20.8%) had undergone any training about antibiotic use or prescribing in COVID-19 patients (Table 2).

**TABLE 2:** Demographic and information of study participants (*n* = 387).

Variable	Frequency (*n*)	Percentage (%)
Gender		
Male	153	39.5
Female	234	60.5
Age (years)		
< 25	11	2.8
25–35	328	84.8
36–45	44	11.4
46–55	4	1.0
Total-experience (years)		
< 5	219	56.6
5–10	137	35.4
>10	31	8.0
Care setting		
Tertiary care	290	74.9
Secondary care	97	25.1
Bed capacity		
< 400	136	35.1
>400	251	64.9
Specialty		
COVID Ward	80	20.7
Emergency	20	5.2
General Medicine	148	38.2
Gynaecology	10	2.6
ICU	45	11.6
Paediatric	9	2.3
Pulmonology	40	10.3
Surgery	35	9.0
Local guidelines/protocols for antibiotic treatment of COVID-19 patients		
Yes	96	24.8
No	268	69.3
Unsure	23	5.9
Periodic reports on local antibiotic resistance data of COVID-19 patients		
Yes	86	22.2
No	271	70.0
Unsure	30	7.8
Training about antibiotic use in COVID-19 patients		
Yes	78	20.2
No	278	71.8
Unsure	31	8.0

### Perception About Antimicrobial Resistance

The majority of the physicians tend to agree or strongly agree with the four statements about antimicrobial resistance. More than three-fourths of the physicians strongly agree that AMR is a global threat (*n* = 313, 80.9%), compared to less than two-thirds who strongly agree that it is also a considerable problem in the Pakistan community (*n* = 221, 57.1%), Pakistani hospitals (*n* = 245, 63.3%) or in their practicing healthcare institutions (*n* = 234, 60.5%) (Supplementary Table S1). These perceptions are similar irrespective of the physician’s gender (*p* = 0.056), years of experience (*p* = 0.875), hospital setting (*p* = 0.445), and training (*p* = 0.497) (Table 3). Multivariate logistic regression revealed no significant association between years of experience and medical specialty with physicians’ perceptions about global, national, or local antimicrobial resistance amid the COVID-19 pandemic (Table 4).

**TABLE 3:** Physicians’ perception and attitudes towards empirical antimicrobial use in COVID-19 patients, by gender, experience, hospital type and training.

Items	Empirical antimicrobial treatment in COVID-19 patients	*p*-value	Perception about antimicrobial resistance amid COVID-19 pandemic	*p*-value	Confidence about prescribing an antibiotic in COVID-19 patients	*p*-value	Attitude towards measures to improve antibiotic prescribing in COVID19 patients	*p*-value
**Gender**								
Male	14.96 (5.24)	0.492	5.53 (1.99)	0.056	9.71 (2.99)	0.034	19.95 (9.35)	0.139
Female	15.31 (4.68)		5.92 (1.94)		10.41 (3.28)		18.69 (7.30)	
Total experience (years)								
< 5	15.05 (4.69)	0.095	5.72 (1.81)	0.875	10.54 (3.01)	0.016	19.20 (7.91)	0.991
5–10	14.69 (5.22)		5.83 (2.11)		9.63 (3.10)		19.20 (8.47)	
>10	17.00 (4.77)		5.77 (2.36)		9.48 (4.29)		19.00 (9.01)	
Care setting								
Tertiary care	15.04 (4.71)	0.375	5.72 (1.91)	0.445	10.34 (3.25)	0.024	19.35 (8.17)	0.483
Secondary care	15.56 (5.45)		5.89 (2.11)		9.50 (2.89)		18.68 (8.26)	
Periodic reports on local antibiotic resistance data of COVID-19 patients								
Yes	15.23 (4.48)	0.897	5.85 (1.68)	0.497	10.98 (2.92)	0.002	16.38 (5.14)	0.001
No	15.12 (5.02)		5.78 (2.07)		9.84 (3.18)		19.77 (8.53)	
Training about antibiotic use in COVID-19 patients								
Yes	15.45 (5.03)	0.645	5.55 (1.87)	0.176	10.08 (3.57)	0.707	19.67 (8.96)	0.217
No	15.16 (4.80)		5.89 (2.01)		10.23 (3.05)		18.42 (7.56)	
Specialty								
COVID Ward	15.05 (4.87)	0.692	5.75 (1.61)	0.806	10.25 (2.94)	0.074	16.82 (5.58)	0.001
Emergency	15.95 (6.29)		6.30 (2.32)		9.55 (3.19)		19.60 (8.72)	
General	15.08 (4.78)	5.75 (2.09)	10.74 (9.42)	19.56 (8.36)
Medicine								
ICU	14.84 (4.65)	5.58 (1.81)	9.42 (2.81)	20.44 (9.24)
Respiratory	15.92 (4.19)	5.95 (2.02)	9.90 (3.45)	14.75 (1.14)
Surgery	14.20 (5.55)	5.69 (2.07)	9.46 (2.82)	20.19 (9.02)

Note: Gynecology and Pediatric specialty was excluded due to small numbers. Empirical antimicrobial treatment in COVID-19, patients = 6 (strongly agree)—30 (strongly disagree).

Perception about antimicrobial resistance amid COVID-19, pandemic = 4 (strongly agree)—20 (strongly disagree).

Confidence about prescribing an antibiotic in COVID-19, patients = 7 (very confident)—28 (very unconfident).

Attitude towards measures to improve antibiotic prescribing in COVID19 patients = 9 (strongly agree)—45 (strongly disagree).

**TABLE 4:** Logistic regression examining the association of years of experience and specialty with physicians’ perception, attitudes, and confidence about antimicrobials.

Items	Empirical antimicrobial treatment in COVID-19 patients OR (95%CI)	*p*-value	Perception about antimicrobial resistance amid COVID-19 pandemic OR (95%CI)	*p*-value	Confidence about prescribing an antibiotic in COVID-19 patients OR (95%CI)	*p*-value	Attitude towards measures to improve antibiotic prescribing in COVID19 patients OR (95%CI)	*p*-value
**Years of experience**								
>10	1.0		1.0		1.0		1.0	
5–10	0.52 (0.22–1.24)	0.138	1.26 (0.51–3.15)	0.616	2.18 (0.52–9.09)	0.286	2.17 (0.84–5.63)	0.112
<5	0.51 (0.22–1.19)	0.118	0.92 (0.38–2.27)	0.862	4.33 (0.96–19.61)	0.057	2.06 (0.81–5.24)	0.127
Specialty								
COVID Ward	1.0		1.0		1.0		1.0	
Emergency	0.72 (0.32–1.65)	0.441	0.82 (0.33–2.03)	0.668	0.51 (0.22–1.18)	0.115	1.53 (0.68–3.45)	0.301
General Medicine	1.37 (0.51–3.68)	0.531	1.51 (0.52–4.42)	0.451	0.82 (0.28–2.45)	0.727	2.80 (1.02–7.72)	0.046
ICU	0.94 (0.54–1.64)	0.839	1.02 (0.56–1.86)	0.946	1.16 (0.66–2.04)	0.606	1.92 (1.09–3.37)	0.023
Respiratory	1.30 (0.62–2.71)	0.419	1.15 (0.52–2.54)	0.725	0.40 (0.18–0.88)	0.024	1.97 (0.93–4.17)	0.076
Surgery	2.36 (1.07–5.21)	0.034	1.13 (0.49–2.64)	0.769	0.67 (0.30–1.51)	0.336	0.49 (0.20–1.22)	0.128

Median split method was used to categorize into agreement and disagreement or confident and not confident categories. Agreement was reference category. Backward LR stepwise approach with Hosmer and Lemeshow test was used to select the model. Gender, age, years of experience, care setting, bed capacity and specialty were entered in the model.

### Using Empirical Antimicrobial Treatment in COVID-19 Patients

In response to questions asking them whether they should use empirical antimicrobial treatment with activity against the specific pathogens, most of the physicians strongly agree or agree that empiric antibiotic treatment is needed for the treatment of *Staphylococcus aureus*, methicillin-resistant (79.4%), followed by *Pseudomonas aeruginosa* (71.3%) and *Staphylococcus aureus*, methicillin-susceptible (62.5%) in COVID-19 patients (Supplementary Table S1). These findings are similar irrespective of the physician’s gender (*p* = 0.492), years of experience (*p* = 0.095), hospital setting (*p* = 0.375), and training (*p* = 0.897) (Table 3). Multivariate logistic regression revealed no significant associations except for surgery. The odds of disagreement by physicians in surgery with empirical antimicrobial treatment in COVID-19 patients (OR 2.36, 95% CI: 1.07–5.21) was higher than physicians in the COVID ward (Table 4).

### Physicians’ Attitudes About Antimicrobials Prescribing Process in COVID-19 Patients

In response to questions asking physicians about the antimicrobial prescribing process in the last month, less than half of the physicians (*n* = 178, 46.0%) said they would consult local guidelines for prescribing antibiotics, and less than a quarter (*n* = 91, 23.5%) said that they would consult local antibiotic resistance data (Supplementary Table S1). However, more than half of the respondents had consulted national guidelines to decide on antimicrobials in COVID-19 patients (*n* = 211, 54.5%).

**FIGURE 1:**
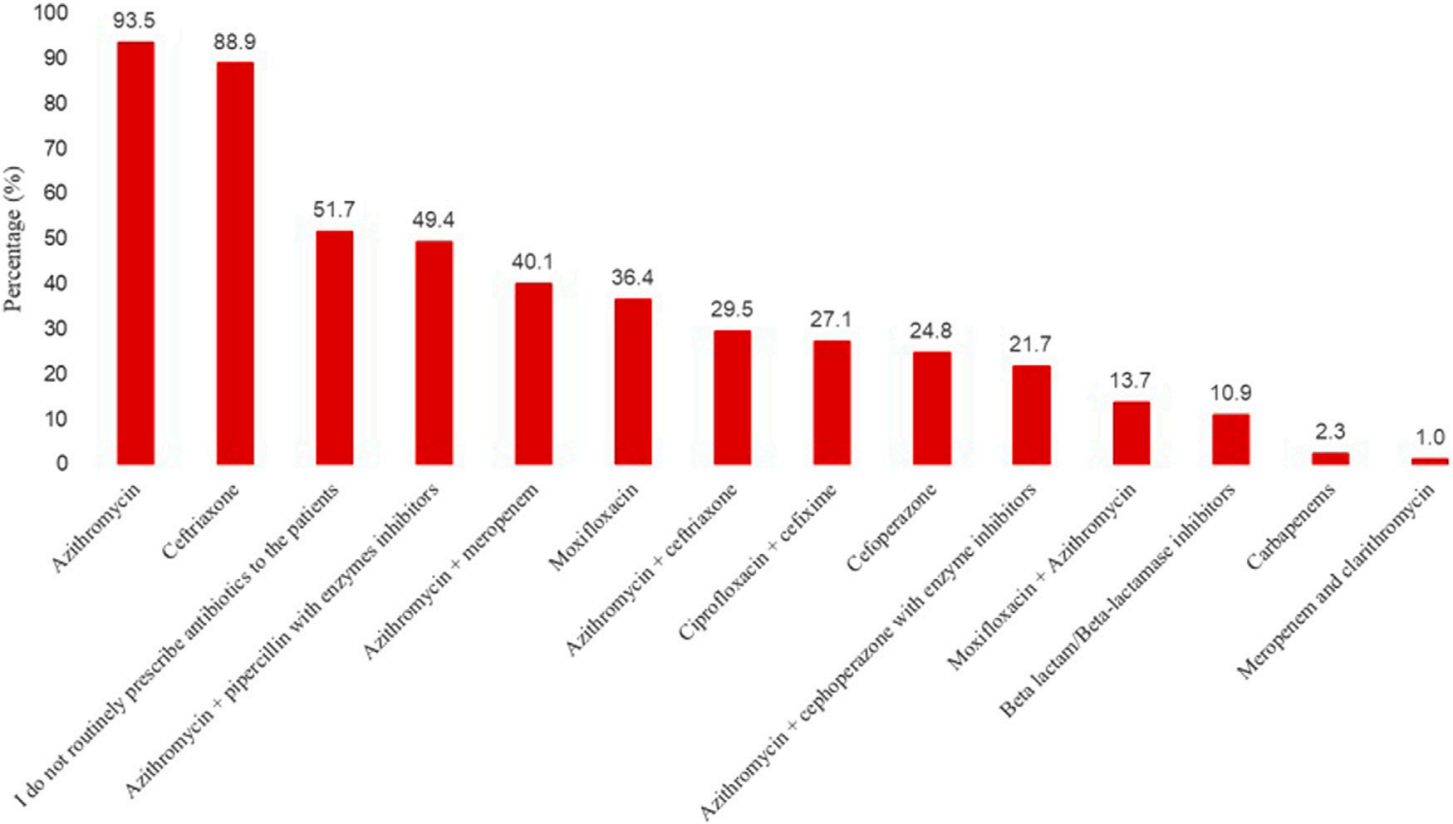
Most usual empirical choice(s) of antibiotics in patients with COVID-19 on the ward.

In response to a question on their usual empirical choice(s) of antibiotics in patients with COVID-19 on the ward, just over half of the physicians stated that they do not routinely prescribe antibiotics to the patients in the ward (Figure 1). Nevertheless, a large number of physicians said that the most usual empirical candidates of antibiotics in patients with COVID-19 include azithromycin (93.5%), ceftriaxone (88.9%), followed by azithromycin plus piparcalin with enzyme inhibitors (49.4%), and azithromycin with meropenem (40.1%).

Physicians with higher years of experience consulted the local guidelines more than physicians with less experience (Table 5). The majority of physicians with more than 10 years of experience consulted local and national guidelines and consulted local resistance data to select empirical antibiotic therapy. Multivariate logistic regression revealed that physicians with less than 10 years of experience had higher odds of not consulting local guidelines for antibiotic therapy (5–10 years: 4.79, 95% CI 1.68–13.66, *p* = 0.003 and < 5 years: 4.71 95% CI 1.62–13.73, *p* = 0.004) compared with physicians with more than 10 years of experience. Similar trends were found for consulting national guidelines and local resistance data to select an empiric antibiotic therapy. In terms of specialty, no significant association was observed except for surgery where the odds of consulting local guidelines by physicians (0.37, 95% CI: 0.59–0.87) and checking local resistance data (0.20, 95% CI: 0.08–0.53) were higher compared with COVID ward (Table 5).

**TABLE 5:** Logistic regression examining the association between years of experience and specialty with antibiotic prescribing process in COVID-19 patients.

Items	In the last month, have you personally used or consulted local guidelines for the therapy of infections when considering an antibiotic for a COVID-19 patient? OR (95% CI)			In the last month, have you personally used or consulted national guidelines for the therapy of infections when considering an antibiotic for a COVID-19 patient? OR (95% CI)			In the last month, have you personally consulted reports on local resistance data to select an empiric antibiotic therapy for a COVID-19 patient? OR (95% CI)		
	Yes	No	*p*-value	Yes	No	*p*-value	Yes	No	*p*-value
Years of experience									
>10	1.0	1.0		1.0	1.0		1.0	1.0	
5–10		4.79 (1.68–13.66)	0.003		4.80 (0.92–25.15)	0.063		2.99 (1.12–8.00)	0.029
< 5		4.71 (1.62–13.73)	0.004		5.77 (1.19–28.02)	0.030		4.35 (1.54–12.26)	0.005
Specialty									
COVID Ward	1.0	1.0		1.0	1.0		1.0	1.0	
Emergency		0.70 (0.30–1.63)	0.412		1.15 (0.49–2.66)	0.748		1.58 (0.49–5.08)	0.441
General Medicine		2.11 (0.60–7.39)	0.242		1.86 (0.60–5.82)	0.284		3.42 (0.38–30.38)	0.270
ICU		0.76 (0.43–1.36)	0.356		0.99 (0.56–1.76)	0.973		1.09 (0.54–2.21)	0.811
Respiratory		0.74 (0.34–1.61)	0.446		1.18 (0.53–2.59)	0.684		1.68 (0.60–4.74)	0.324
Surgery		0.37 (0.59–0.87)	0.022		0.58 (0.24–1.42)	0.232		0.20 (0.08–0.53)	0.001

Logistic regression was used: Backward LR stepwise approach with Hosmer and Lemeshow test was used to select the most relevant model. Gender, age, years of experience, care setting, bed capacity and specialty were entered in the model.

### Confidence About Prescribing an Antibiotic in COVID-19 Patients

Regarding how confident they feel when prescribing an antibiotic, the physicians demonstrated a high level of confidence in prescribing antibiotics among COVID-19 patients. For example, more than half of the physicians were very confident about making an accurate diagnosis of infection (*n* = 240, 62.0%%) and interpreting microbiological results (*n* = 199, 51.4%). Likewise, the physicians were confident enough to select an appropriate antibiotic (*n* = 229, 59.2%) coupled with the correct dose of antibiotics in COVID-19 patients (*n* = 219, 56.6%). Furthermore, 240 (62.0%) physicians said they do not prescribe an antibiotic if they are unsure about their diagnosis (Supplementary Table S3). In addition, male physicians (*p* = 0.034), a physician with more than 10 years of experience (*p* = 0.016), physicians working in secondary care (*p* = 0.024), and physicians who consulted or used local or national guidelines (*p* = 0.001) were reported to have a significantly higher level of confidence than their counterparts (Table 4). Multivariate logistic regression revealed no association of physicians’ confidence with years of experience and different specialties except for respiratory medicine, where physicians are more confident about prescribing antibiotics than physicians in the COVID-19 ward (Table 4).

### Measures to Improve Antibiotic Prescribing in COVID-19 Patients

In response to the statement about measures to improve antibiotic prescribing in COVID-19 patients, many physicians were agreed with most of the measures that could help improve antibiotic prescribing among COVID-19 patients (Table 3). For example, more than three-quarters of the physicians believed that advice from a senior colleague (n = 336, 86.8%), infectious disease (ID) physician (*n* = 315, 81.4%), and a microbiologist (*n* = 291, 75.2%) could facilitate rational prescribing of antibiotics in COVID-19 patients nevertheless they were less in agreement to consider the advice of a pharmacist (*n* = 230, 59.4%). Many physicians held a positive attitude towards the implementation of antimicrobial stewardship programs (*n* = 302, 78.0%) and availability of local treatment guidelines (*n* = 298, 77.0%) coupled with periodic antibiotic resistance information (*n* = 304, 78.6%). Those who consulted or used local or national guidelines reported significantly higher trust in taking advice from relevant professionals (*p* = 0.001). Multivariate logistic regression revealed a similar attitude towards improving antibiotic prescribing in COVID-19 patients between physicians with different years of experience. Physicians in emergency and general medicine had higher odds of having disagreement compared with physicians COVID ward for items measuring attitude towards measures to improve antibiotic prescribing in COVID-19 patients (Table 4).

## Discussion

The present study demonstrates a high awareness about AMR among physicians and reported a positive attitude to minimize the AMR risk among COVID-19 patients. Besides, physicians were confident about the antibiotic prescribing in patients with COVID-19 infection.

The majority of physicians were aware that AMR is a global problem affecting the Pakistani community and hospitals. The previous literature shows that AMR is continuously escalating daily worldwide in both hospital and community settings. For example, more than three-quarters of the physicians working in Nigerian hospitals considered AMR a global issue and a local concern (Babatola et al., 2021). Our previous studies conducted in Pakistan have also highlighted that physicians have rightly understood the issue of AMR in Pakistani hospitals and communities (Hayat et al., 2019a; Hayat et al., 2019b; Hayat et al., 2020). Therefore, it is critical to raise awareness of AMR, particularly during COVID-19. As indicated in several studies, the use of antibiotics among COVID-19 patients has grown significantly, which could surge the AMR risk (Rawson et al., 2020a; Rawson et al., 2020b; Li et al., 2020; Ukuhor, 2021).

Most of our study respondents (49.9%) said they had not consulted local treatment guidelines while prescribing antibiotics to COVID-19 patients, which seems alarming and could potentiate AMR burden. Surprisingly, only 23.5% of respondents used local resistant data to prescribe antibiotics even though the availability of resistance patterns is crucial for optimal decision-making regarding antibiotic use. Most hospital settings lack treatment guidelines to manage infectious diseases in Pakistan, and physicians consult international guidelines. This problem has been highlighted in our previous studies (Hayat et al., 2019a; Mustafa et al., 2021). This is true that a lack of local guidelines to manage COVID-19 patients could surge irrational antibiotic utilization, which will potentially worsen the AMR problem.

The physicians in our study (79.4%) were prescribing antibiotics empirically in COVID-19 patients infected with *Staphylococcus aureus, methicillin-resistant infections*. The COVID-19 patients are at a high risk of catching secondary infections of bacterial-viral origin (Spoto et al., 2020). Therefore, it is necessary to cope with these infections by using antimicrobials, including antibiotics. In addition, previous studies have shown that risk or mortality due to *Staphylococcus aureus* infections could be significantly enhanced among COVID-19 patients (Hughes et al., 2020; Spoto et al., 2020; Russell et al., 2021).

Azithromycin (93.5%) and ceftriaxone (88.9%) were the most frequently prescribed medications by our study physicians to treat coinfections in COVID-19 patients. Azithromycin was initially repurposed for COVID-19 (Butler et al., 2021). These drugs were commonly prescribed to treat numerous infections, including community and hospital-acquired pneumonia, which could also occur in COVID-19 patients. Therefore, it is reasonable to believe that using azithromycin and ceftriaxone could have increased in hospital settings during the pandemic. Similar findings have been reported previously (Nestler et al., 2020; Chedid et al., 2021). Azithromycin possesses antiviral potential by multiplying interferons and limiting virus amplification; however, its routine use is not recommended in suspected COVID-19 older adults (Mohanta et al., 2020; Butler et al., 2021).

Most of our study physicians were confident about infection diagnosis and antibiotic prescribing with correct dose, route, and duration among COVID-19 patients. Nevertheless, this high confidence could be compromised due to a lack of treatment guidelines, inadequately qualified professionals, and inadequate diagnostic facilities in Pakistan (Hayat et al., 2019a).

Many physicians favored implementing ASPs in Pakistan to cut the risk of AMR and irrational prescribing of antimicrobials in COVID-19 patients. The effectiveness of ASPs has already been reported in the literature regarding lowering AMR, cost of therapy and irrational antimicrobials use (MacDougall and Polk, 2005; Lai et al., 2016; Hughes and Moore, 2019). Furthermore, the importance of ASPs has significantly increased during the ongoing COVID-19 outbreak (Mazdeyasna et al., 2020; Ashiru-Oredope et al., 2021; Kubin et al., 2021). However, the implementation of ASPs in Pakistani hospitals is still emerging, and not all hospitals have implanted these programs. In addition, previous studies have shown that healthcare professionals are not familiar with ASPs, and their experience in such types of facilities is also limited (Hayat et al., 2019a; Hayat et al., 2020). Thus, there is a pressing need for the government to make major efforts to implement ASPs throughout all healthcare settings.

More than 80% of physicians considered advice from an ID physician to be one of the appropriate strategies to improve antimicrobial prescribing in COVID-19 patients. This has already been documented that ID physician-led antimicrobial prescribing helps minimize consumption and antibiotic resistance with the best possible clinical outcomes (Matono et al., 2021). Furthermore, the interprofessional collaboration between physicians, pharmacists, and nurses is essential for the smooth running of the healthcare system and improving patient outcomes that is missing in Pakistani hospitals (Hayat et al., 2019a; Hayat et al., 2021).

There are several limitations associated with this study. First, this research was carried out in a single province in Pakistan with convenience sampling; therefore, its results have limited generalizability. Nonetheless, this province has more than 50% of the total population of Pakistan, and its healthcare facilities are exemplary for other provinces of Pakistan. Second, the respondents of this study were physicians working only in public hospitals, and the views of physicians practicing in private hospitals and clinics are unknown. Third, this study has not assessed the antimicrobial prescribing practices of physicians in COVID-19 patients. Despite these limitations, this study is the first to present an insight of physicians about antimicrobial resistance and antibiotic prescribing among COVID-19 patients.

## Conclusion

The physicians were well aware of the problem of AMR. Nonetheless, not many physicians consulted local antibiotic resistance information to prescribe antibiotic therapy empirically in COVID-19 patients. However, most physicians were confident to select the appropriate antibiotic with the correct dose, route, and duration. Implementation of ASPs coupled with advice from a senior colleague and ID physicians were the main approaches suggested by physicians to help antimicrobial prescribing and cut AMR risk among COVID-19 patients. There is an urgent need to launch stewardship programs targeted at optimum antibiotic utilization supported by regional and institution-based standard treatment guidelines in patients with COVID-19 infection to minimize antibiotic resistance amplification. Massive efforts are required to help vaccinate the public at the mass level to limit COVID-19 risk that could help cut irrational antibiotic use among COVID-19 patients.

## Data Availability

The original contributions presented in the study are included in the article/Supplementary Material, further inquiries can be directed to the corresponding author.
