# Supplementary material for: Perception, Attitude, and Confidence of Physicians About Antimicrobial Resistance and Antimicrobial Prescribing Among COVID-19 Patients: A Cross-Sectional Study From Punjab, Pakistan
Source: Front Pharmacol. 2022 Jan 4;12:794453. doi: 10.3389/fphar.2021.794453 (PMC8763689; doi:10.3389/fphar.2021.794453)
Supplement: Supplementary file 1 [file Table1.DOCX]

Supplementary Tables

**Table S1**. Physicians’ opinion about the empirical antimicrobial treatment, antimicrobial resistance and measures to improve antimicrobial prescribing in COVID-19 patients

| **Question** | **SA** | **A** | **N** | **D** | **SD** |  |
| --- | --- | --- | --- | --- | --- | --- |
| **Perception about antimicrobial resistance amid COVID-19 pandemic** | | | | | | |
| Antimicrobial resistance is a global problem | 313 (80.9) | 71 (18.3) | 1 (0.3) | 2 (0.5) | 0 (0.0) |  |
| Antimicrobial resistance is a problem in Pakistani community | 221 (57.1) | 105 (27.1) | 41 (10.6) | 11 (2.8) | 9 (2.3) |  |
| Antimicrobial resistance is a problem in Pakistani hospital | 245 (63.3) | 120 (31.0) | 14 (3.6) | 5 (1.3) | 3 (0.8) |  |
| Antimicrobial resistance is a problem in your hospital | 234 (60.5) | 139 (35.9) | 9 (2.3) | 4 (1.0) | 1 (0.3) |  |
| **We should use** **empirical antimicrobial treatment with activity against the following pathogens** | | | | | | |
| Staphylococcus aureus, methicillin susceptible | 184 (47.5) | 58 (15.0) | 50 (12.9) | 67 (17.3) | 28 (7.2) |  |
| Staphylococcus aureus, methicillin resistant | 246 (63.6) | 61 (15.8) | 30 (7.8) | 36 (9.3) | 14 (3.6) |  |
| Pseudomonas aeruginosa | 200 (51.7) | 76 (19.6) | 45 (11.6) | 41 (10.6) | 25 (6.5) |  |
| Atypical pathogens | 124 (32.0) | 53 (13.7) | 71 (18.3) | 95 (24.5) | 44 (11.4) |  |
| Candida spp. | 69 (17.8) | 30 (7.8) | 83 (21.4) | 144 (37.2) | 61 (15.8) |  |
| Aspergillus spp. | 68 (17.6) | 27 (7.0) | 87 (22.5) | 144 (37.2) | 61 (15.8) |  |
| **Physicians attitude towards measures to improve antibiotic prescribing in COVID19 patients** | | | | | | |
| Advice from a senior colleague | 189 (48.8) | 147 (38.0) | 25 (6.5) | 11 (2.8) | 15 (3.9) |  |
| Advice from an infectious disease specialist | 183 (47.3) | 132 (34.1) | 25 (6.5) | 23 (5.9) | 24 (6.2) |  |
| Advice from a microbiologist | 159 (41.1) | 132 (34.1) | 46 (11.9) | 24 (6.2) | 26 (6.7) |  |
| Advice from a pharmacist | 84 (21.7) | 146 (37.7) | 78 (20.2) | 42 (10.9) | 37 (9.6) |  |
| Implementation of antimicrobial stewardship programs | 131 (33.9) | 171 (44.2) | 32 (8.3) | 27 (7.0) | 26 (6.7) |  |
| Availability of locally developed guidelines for therapy of infections | 138 (35.7) | 160 (41.3) | 33 (8.5) | 29 (7.5) | 27 (7.0) |  |
| Availability of systematic reports about resistance data | 135 (34.9) | 169 (43.7) | 28 (7.2) | 32 (8.3) | 23 (5.9) |  |
| Implementation of monitoring systems of used antibiotics | 124 (32.0) | 130 (33.6) | 44 (11.4) | 58 (15.0) | 31 (8.0) |  |
| Computer-aided prescribing | 113 (29.2) | 129 (33.3) | 46 (11.9) | 58 (15.0) | 41 (10.6) |  |

SA: strongly agree, A: agree, N: neutral, D: disagree, SD: strongly disagree

**Table S2.** Antibiotic prescribing process in COVID-19 patients by year of experience

| **Questions** | **<5 years** | **6-10 years** | **>10 years** | **p-value** |
| --- | --- | --- | --- | --- |
| **In the last month, have you personally used or consulted local guidelines for the therapy of infections when considering an antibiotic for a COVID-19 patient? (** | | | | |
| Yes | 92 (44.2) | 62 (47.0) | 24 (77.4) | 0.002 |
| No | 116 (55.8) | 70 (53.0) | 7 (22.6) |  |
| **In the last month, have you personally used or consulted national guidelines for the therapy of infections when considering an antibiotic for a COVID-19 patient?** | | | | |
| Yes | 116 (55.5) | 71 (52.6) | 24 (80.0) | 0.022 |
| No | 93 (44.5) | 64 (47.4) | 6 (20.0) |  |
| **In the last month, have you personally consulted reports on local resistance data to select an empiric antibiotic therapy for a COVID-19 patient?** | | | | |
| Yes | 49 (29.2) | 26 (24.5) | 16 (57.1) | 0.003 |
| No | 119 (70.8) | 80 (75.5) | 12 (42.9) |  |

p-value was obtained by Chi-Sq test; excluded ‘unsure’ responses; % within year of experience

**Table S3**. Physicians’ confidence about prescribing an antibiotic in COVID-19 patients

| **Questions** | **Very confident** | **Confident** | **Unconfident** | **Very Unconfident** |
| --- | --- | --- | --- | --- |
| Making an accurate diagnosis of infection | 240 (62.0) | 142 (36.7) | 5 (1.3) | 0 (0.0) |
| Deciding not to prescribe an antibiotic if you are not sure about your diagnosis | 240 (62.0) | 140 (36.2) | 7 (1.8) | 0 (0.0) |
| Choosing the correct antibiotic | 229 (59.2) | 153 (39.5) | 4 (1.0) | 1 (0.3) |
| Choosing the correct dose and interval of administration | 219 (56.6) | 165 (42.6) | 2 (0.5) | 1 (0.3) |
| Choosing between intravenous and oral administration | 207 (53.5) | 177 (45.7) | 2 (0.5) | 1 (0.3) |
| Interpreting microbiological results | 199 (51.4) | 183 (47.3) | 3 (0.8) | 2 (0.5) |
| Planning the duration of the antibiotic treatment | 200 (51.7) | 182 (47.0) | 4 (1.0) | 1 (0.3) |
